# That brachycephalic look: Infant-like facial appearance in short-muzzled dog breeds

**DOI:** 10.1017/awf.2022.6

**Published:** 2023-01-26

**Authors:** Elizabeth S Paul, Rowena MA Packer, Paul D McGreevy, Emily Coombe, Elsa Mendl, Vikki Neville

**Affiliations:** 1 Bristol Veterinary School, University of Bristol, Langford House, Langford BS40 5DU, UK; 2 Department of Clinical Sciences and Services, The Royal Veterinary College, Hatfield, Herts AL9 7TA, UK; 3 School of Environmental and Rural Science, Faculty of Science, Agriculture, Business and Law, University of New England, Armidale, NSW 2351, Australia; 4 Positive Dog Training, Long Ashton, Bristol, UK

**Keywords:** animal welfare, brachycephalic, breed, cute, dog, ‘kindchenschema’

## Abstract

Brachycephalic dog breeds are highly popular, yet their conformation-related disorders represent a major welfare concern. It has been suggested that the current popularity of such breeds can be explained by their cute, infant-like facial appearances. The concept of ‘kindchenschema’ refers to the observation that certain physical features of infant humans and other animals can automatically stimulate positive and nurturant feelings in adult observers. But the proposal that brachycephalic dogs possess heightened ‘kindchenschema’ facial features, even into adulthood, has never been formally investigated. Here, we hypothesised that relative muzzle shortening across a range of breeds would be associated with known ‘kindchenschema’ facial features, including a relatively larger forehead, larger eyes and smaller nose. Relative fronto-facial feature sizes in exemplar photographs of adult dogs from 42 popular breeds were measured and associated with existing data on the relative muzzle length and height-at-withers of the same breeds. Our results show that, in adulthood, shorter-muzzled breeds have relatively larger (taller) foreheads and relatively larger eyes (i.e. area of exposed eyeball relative to overall face area) than longer-muzzled breeds, and that this effect is independent of breed size. In sum, brachycephalic dog breeds do show exaggeration of some, but not all, known fronto-facial ‘kindchenschema’ features, and this may well contribute to their apparently cute appearance and to their current popularity as companion animals. We conclude that the challenge of addressing conformation-related disorders in companion dogs needs to take account of the cute, ‘kindchenschema’ looks that many owners are likely to be attracted to.

## Introduction

Brachycephalic dogs, with their flattened faces and shortened muzzles, have long been popular pets (Skipper 2021). But the unprecedented rise in ownership of breeds such as the Pug, Boston terrier and French bulldog in recent years has prompted numerous researchers and organisations to voice concerns about the health and welfare consequences of such facial morphologies (Asher *et al.* 2009; Rooney & Sargan 2009; Bateson 2016; Brachycephalic Working Group 2018; British Veterinary Association 2020a,b). Discourse has focused largely on a single, central conflict: between the clinical problems associated with extreme muzzle shortening (O’Neill *et al.* 2015, 2017, 2019, 2020; Packer *et al.* 2015a,b; Packer & Tivers 2015; Liu *et al.* 2017; Seppanen *et al.* 2019; Fawcett *et al.* 2019) and public demand for extremely brachycephalic breeds, not least because of their putatively appealing, cute appearance (Packer *et al.* 2019, 2020). Recent research has indicated that many owners understand that brachycephalia can have adverse consequences for health but choose to keep such breeds anyway (Packer *et al.* 2020). This may be partly due to the perceived positive behavioural traits of some of these breeds (Packer *et al.* 2020), with owners’ denial of their dogs’ health problems (Packer *et al.* 2012), or with the paradoxical appeal of disabled dogs (Sandøe *et al.* 2017; Serpell 2019). Either way, there is little doubt that some aspects of the appearance of brachycephalic animals is also a crucial driver of ownership (e.g. Waller *et al.* 2013; Packer *et al.* 2017, 2020).

To explore why brachycephalic dogs are so popular, the current study was designed to investigate the precise nature of the ‘brachycephalic look’ in contemporary dog breeds. Commentators and researchers appear to agree that such breeds carry the appealing facial features associated with human perceptions of cuteness, and that these features are a key determinant of the popularity of short-muzzled dogs (e.g. Serpell 2002; McGreevy *et al.* 2013; Packer *et al.* 2017; Sandøe *et al.* 2017; O’Neill *et al.* 2022). But these claims have never been formally investigated. Do brachycephalic dogs really look cute? And if so, in what ways?

**Figure 1.**
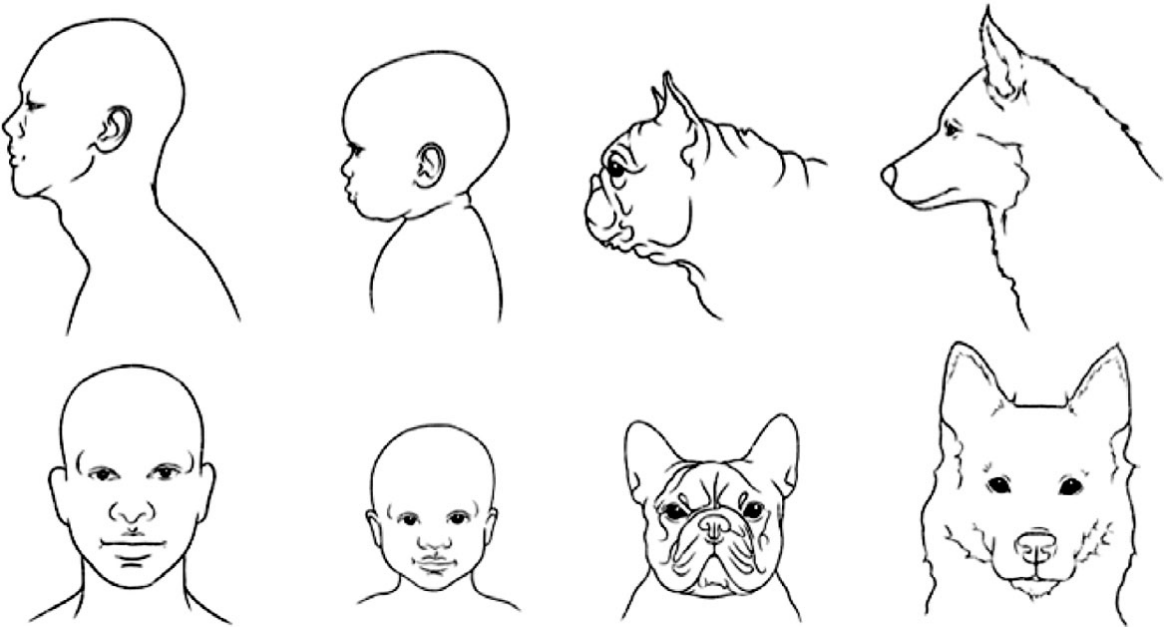
Sagittal and coronal plane line-drawings of (from left to right): adult human, infant human, adult French bulldog and adult Siberian husky.

The focus of this paper is the ‘kindchenschema’ effect (also known as the cute effect); a well-studied phenomenon in psychological research in which infantile features in images of both children and animals are regularly found to provoke positive and affectionate feelings in human observers, and to prompt careful and nurturant behaviours (for a recent review, see Kringelbach *et al.* 2016). Our aim here is to discover whether relative muzzle shortening across a range of dog breeds is linked to the key facial features known to be appealing to, and to elicit nurturant behaviours in, human observers. Specifically, we sought to discover whether muzzle shortening in the *sagittal* plane of dogs’ heads (i.e. in profile) is statistically associated with known ‘kindchenschema’ features in the *coronal* plane (i.e. fronto-facial features such as the relative size of eyes and forehead) (see Figure 1). To do this, we investigated the links between two datasets: (i) pre-existing anatomical measures of relative muzzle shortening across a range of popular dog breeds; and (ii) photograph-based measures of fronto-facial features of the same breeds, collected for this study. We hypothesised that shorter relative muzzle length in the sagittal profiles of breeds would be linked with more infantile feature proportions in the frontal (coronal) planes of their faces.

### The ‘kindchenschema’ effect

People’s perceptions of cuteness (i.e. of pleasant and appealing youthfulness) are based upon the detection of a wide range of features associated with infancy, including various aspects of body shape, voice, skin texture, hair colour and movement style (Berry & McArthur 1986; Kringelbach *et al.* 2016). However, the infantile features of greatest influence are in the face. The cranium of newborn humans is relatively larger than the midface, and grows more slowly with age, leading to a distinctive pattern of change in the both the sagittal and coronal planes of humans’ heads as they develop from infancy to adulthood (Pittenger & Shaw 1975; Berry & McArthur 1986). As a result, the facial appearance of infants is distinctively different from that of adults, with babies exemplifying relatively larger eyes (i.e. the area of the orbit that is visible), smaller noses and taller, more protruding foreheads (Lorenz 1943, 1971; see also Berry & McArthur 1986; Kringelbach *et al.* 2016). Early studies of the effects of these ‘kindchenschema’ facial features demonstrated that youthful variants of line-drawn sagittal-plane images, with relatively smaller, lower- and mid-face regions, can heighten people’s cuteness perceptions of human children, animals, and even of cartoon depictions of inanimate objects (Gardner & Wallach 1965; Alley 1981; Berry & McArthur 1985). That said, the vast majority of psychological research into ‘kindchenschema’ and their effects on adult observers has focused on the coronal plane of the head (fronto-facial features: Fullard & Reiling 1976; Sternglanz *et al.* 1977; Hildebrandt & Fitzgerald 1979; Alley 1981; Berry & McArthur 1985; McKelvie 1993; Brosch *et al.* 2007; Glocker *et al.* 2009; Parsons *et al.* 2011; Senese *et al.* 2013; Esposito *et al.* 2015; Almanza-Sepdulveda *et al.* 2018). For example, images manipulated to convey more infant-like features, such as large, rounded eyes and small, ‘button’ noses are rated as more attractive, more cute, and induce more careful and care-giving behaviour in observers, than those with more adult forms. These sorts of associations between coronal-plane infantile morphology and cuteness attributions among adult care-givers, do not occur by chance. Human reproduction is thought to depend, at least to some extent, on the ‘kindchenschema’ effect, aiding the maximisation of care-giving to younger offspring (Kringlebach *et al.* 2016). And, given that the front of the face is the key point of focus for human social and emotional perception processing, it is not surprising that fronto-facial infantile features represent prime releasing stimuli for such behaviour (e.g. Lacruz *et al.* 2019), with eye-tracking studies indicating that visual attention to this part and plane of the face is central to human cuteness perceptions (Borgi *et al.* 2014).

### ‘Kindchenschema’ in dogs: Sagittal plane, coronal plane, or both?

The changes that occur during cranio-facial growth in human infants are mirrored across numerous species. As a result, the young of many animals can readily generate ‘kindchenschema’ effects among human observers, and experimentally enhancing images of animals’ facial features in an infantile manner — e.g. enlarging the eyes or forehead — can magnify these effects (e.g. Archer & Monton 2011; Lehmann *et al.* 2013; Chersini *et al.* 2018). There is also evidence that similar processes persist in generating both human- and animal-focused ‘kindchenschema’ effects (Golle *et al.* 2013).

As noted above, most experimental investigations of the influence of infantile morphology on cuteness perceptions, in both animal and human images, have focused upon features in the coronal plane of the face, although sagittal plane manipulations have also been found to also generate ‘kindchenschema’ effects (Gardner & Wallach 1965; Alley 1981; Berry & McArthur 1985). The facial profiles of brachycephalic dog breeds are noteworthy because their adult heads look somewhat infantile — sharing some of the features of both puppies and human infants, with a relatively small, non-protruding mid-face and more prominent forehead (see Figure 1). So sagittal-plane features such as these may be all that is needed for people to perceive brachycephalic dogs as cute (e.g. Serpell 2002; Serpell & Paul 2011). However, another possibility is that short muzzles in dogs are also associated with infantile facial features in the coronal plane of the face, and that short muzzles may contribute to people’s perceptions of cuteness in brachycephalic breeds through this route as well. For example, it is possible that many brachycephalic breeds have not only relatively short muzzles, but also relative larger eyes, small noses and large, tall foreheads; i.e. known coronal-plane (fronto-facial) infantile features with strong ‘kindchenschema’ effects.

### Measurement of muzzle length and fronto-facial ‘kindchenschema’ features

In recent years, brachycephalia in the veterinary context has focused on the relative length of the muzzle, which has been investigated using anatomical measurements in the sagittal and transverse planes of affected dogs’ heads (e.g. Georgevsky *et al.* 2014; Packer *et al.* 2015b, based on Sutter *et al.* 2008). To obtain measures of the fronto-facial (‘kindchenschema’) features of different breeds in the coronal plane of the head (i.e. features as would normally be observed by looking directly at a dog’s face), a wide range of photographic images were used here, sourced via the internet. Using these images, the actual ‘looks’ of the dogs, as seen by owners and potential purchasers in the real world, could be established (i.e. incorporating, but not separately measuring, anatomical features including skull morphology, soft tissue thickness, and fur thickness). By using externally sourced photographic images, we were able to access a broad group of breed exemplars. The fronto-facial features considered here were those that have previously been found to be associated with infancy and cuteness attributions. They were those that could also be readily applied to facial measurements, from photographs, in dogs: relative forehead size, relative eye size, relative nose size. Relatively large (taller) foreheads, large eyes, and small noses are all associated with infancy in humans and with ‘kindchenschema’ effects in experimental studies (Sternglanz *et al.* 1977; Hildebrandt & Fitzgerald 1979; Alley 1981; Berry & McArthur 1985; McKelvie 1993; Glocker *et al.* 2009). Additionally, some studies have found that eye shape and degree of eye separation (hypertelorism) can also influence people’s judgements, so these, as well as nose shape, were also included in our measurements (Berry & McArthur 1986; McKelvie 1993; Hecht & Horowitz 2015).

## Materials and methods

### Measures of relative muzzle length

Two continuous measures of relative muzzle length based on existing, published datasets were used here (Georgevsky *et al.* 2014; Packer *et al.* 2015b). Although these measures are fairly similar, we chose to make use of both. This was because some measurement differences exist between the two, and it is possible that these might have an impact on any relationships among muzzle-shortening and fronto-facial features. Most notably, the CFR incorporates forehead-doming into its measure, while the CI does not (Georgevsky *et al.* 2014). The CI is calculated by dividing the anterior-posterior length of the skull by its width at its widest part (×100), producing a measure in which *higher* values represent shorter-muzzled, more brachycephalic, cranial morphologies (McGreevy *et al.* 2004, 2013; Roberts *et al.* 2010; Georgevsky *et al.* 2014). Georgevsky and colleagues (Georgevsky *et al.* 2014) calculated the CI of 960 dogs across 80 breeds (six adult males and six adult females per breed), taking the measurements from standardised images of live dogs photographed from above (transverse plane of head).

The CFR is calculated by using soft tape measurements of live dogs’ head dimensions, without photographs; Dividing the muzzle length by the cranial length produces a measure in which lower values represent shorter-muzzled, more brachycephalic morphologies (Sutter *et al.* 2008). Packer *et al.* (2015a) calculated the CFR for 700 dogs of 97 breeds (varying numbers of exemplars per breed). For the current study, CFR means were calculated using data from dogs of six months of age and above (a majority subset of that used in Packer *et al.* 2015b). We make the assumption here that at six months of age, most dogs have largely adult head-shapes, although this remains to be confirmed empirically.

### Measures of facial ‘look’: Fronto-facial ‘kindchenschema’ features

Twenty face-forward photographs of dogs from each breed considered here were obtained from internet searches using the search term “*breed name* face.” Two different search engines were used to avoid any possible bias associated with search algorithms; ten images per breed were harvested from *Google* and ten from *Bing* (globally, the two most popular search engines at the time of access, in January 2020). The images originated from a range of sources, including private postings, stock photos and commercial websites. Criteria for selecting images were: the whole dog face must be visible and of good quality (i.e. photograph not dark or blurred); the dog must be facing directly at the camera; the face could be tilted to the left or right, but not up/down; the top of the skull, the bottom of the nose and the top of the mouth were all visible; no distorted or manipulated images (e.g. with distortion lenses or obvious photo-shop modifications); no eyes closed; no extreme facial expressions (e.g. barking/growling/attacking); no puppies; no repeated individuals (i.e. none from same website or with identical coat patterns); no extreme or comical grooming styles.

**Figure 2.**
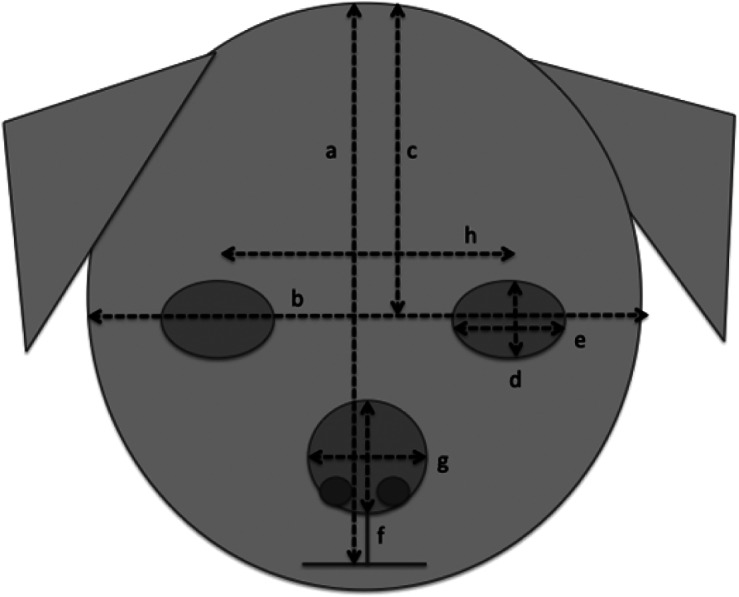
Measurements made on all dog face images, to calculate mean relative feature sizes for each breed: (a) face height; (b) face width; (c) forehead height; (d) eye height; (e) eye width; (f) nose height; (g) nose width; (h) distance between the eyes (to calculate relative distance between the eyes [h]/[b]).

A researcher, blind to the hypotheses of the study, measured the face and eye dimensions of each of the 20 images per breed, using Image J software (https://imagej.nih.gov/ij/). The measures that were taken are illustrated in Figure 2. From these, six variables (means per breed) were calculated: relative eye size (single eye aperture area/total face area), relative nose size (nose area/total face area), relative forehead size (forehead area/total face area), eye height/width ratio (i.e. indicating degree of eye-roundedness); nose height/width ratio (i.e. indicating degree of nose-roundedness); relative eye separation (distance between eyes/total face width). Areas were calculated assuming that eyes, faces and noses were ellipse-shaped (area = π (*H/2)* (W/2)). Details of the means of these measurements for each breed are given in Appendix 1. The full dataset is available from the corresponding author upon reasonable request. It was noted that, despite the wide range of source photographs, considerable within-breed homogeneity in relative feature sizes was found, with high correlations among exemplars of each breed, indicating considerable breed-level conformity among these facial features.

### Measures of breed size

To control for the possible confounding effects of overall breed size on the relationships among sagittal and coronal facial features, measures of estimated breed height were also included in our analyses. UK Kennel Club breed standard descriptions of breed height (including height ranges for male and female exemplars) were averaged to provide estimations of the size of each of the 42 breeds. This was supplemented by American Kennel Club breed standard information where UK estimates were not available (American Kennel Club 2021; The Kennel Club 2021).

### Dog breeds included in the study

Both CI and CFR data are available for 57 breeds in total (Georgevsky *et al.* 2014; Packer *et al.* 2015b). Of these, 15 were excluded from the present study because reasonable measurement of fronto-facial feature size from photographic images was not possible, due to excess hair or fur around the face (breeds excluded: Akita, Bearded collie, Bichon frise, Cairn terrier, Chinese crested, Griffon Bruxellois, Japanese spitz, Leonberger, Maltese, Norwegian elkhound, Old English sheepdog, Pomeranian, Shih tzu, Tibetan terrier, West Highland white terrier). Mean CI and CFR data for each of the remaining 42 breeds studied here are shown in Table 1.

**Table 1.** Relative muzzle length (CFR, CI) of the 42 breeds studied

Breed	CFR	CI
Pug	0.084	95.685
French bulldog	0.182	105.344
Bulldog	0.217	88.722
Boston terrier	0.278	83.273
Boxer	0.324	63.495
Chihuahua (long coat & smooth coat)	0.348	67.442
Cavalier King Charles spaniel	0.395	74.521
Bullmastiff	0.410	80.917
Papillon	0.440	68.990
Hungarian vizsla	0.490	50.659
Mastiff	0.490	93.246
Staffordshire bull terrier	0.508	75.475
Rottweiler	0.515	69.420
Miniature schnauzer	0.540	64.444
Spaniel (cocker)	0.547	44.278
Spaniel (English springer)	0.550	51.594
Retriever (golden)	0.552	58.073
Jack Russell terrier	0.562	64.520
Beagle	0.562	66.487
Rhodesian ridgeback	0.564	45.600
Bernese mountain dog	0.567	59.174
Spaniel (American cocker)	0.580	74.711
Retriever (Labrador)	0.582	74.711
Australian shepherd dog	0.590	49.141
Gordon setter	0.590	52.982
Dalmatian	0.599	49.581
Australian kelpie	0.600	58.899
Dachshund (miniature)	0.617	47.647
Irish setter	0.620	47.647
Bassett hound	0.623	50.517
Welsh corgi (Cardigan)	0.650	54.916
German short-haired pointer	0.660	57.544
Great Dane	0.663	54.074
Border collie	0.665	57.851
Dobermann	0.668	52.681
Greyhound	0.713	47.175
German shepherd dog	0.714	55.536
Weimaraner	0.725	53.630
Saluki	0.730	43.720
Shetland sheepdog	0.790	57.982
Bull terrier	0.800	60.269
Whippet	0.870	47.854

### Statistical analysis

The assumptions of parametric statistics were checked using normality and QQ plots. Pearson product moment correlational analyses were used to investigate associations among the dog breeds’ CI scores, CFR scores and estimated height, and among the fronto-facial measures calculated here (relative eye size, nose size and forehead size; eye and nose height/width ratio; relative eye separation). Following this, multiple linear regressions, using muzzle-shortening score (CFR or CI) and estimated breed size as potential predictor variables, were conducted for all six fronto-facial measures.

## Results

Preliminary analyses showed that CI and CFR were highly correlated (this presented as an inverse correlation as low CFR values and high CI values are indicative of relatively shortened muzzles: *r* = –0.764, n = 42; *P* < 0.001).

Both CFR and CI also showed modest associations with estimated breed height, with smaller breeds in the present sample also having somewhat shorter muzzles. This association reached significance for CFR but not quite for CI, and the *P* < 0.05 level (CFR: *r* = 0.360, n = 42; *P* = 0.019; CI: *r* = –0.304, n = 42; *P* = 0.051).

The fronto-facial variables measured here showed a number of associations. Two key ‘kindchenschema’ measures, relative forehead size and relative eye size, were positively correlated (Table 2). Relative eye size (i.e. aperture area/face area) was also positively correlated with eye height/width ratio. Mean eye height/width ratio across the entire sample was 0.774 (indicating wider-than-tall, almond-shaped eyes for most dogs). These findings show that those breeds with relatively larger eyes also tend to have rounder, less almond-shaped eyes.

**Table 2.** Associations (Pearson product moment correlations) among fronto-facial ‘kindchenschema’ measures

	Relative eye size	Relative nose size	Eye height/width ratio	Nose height/width ratio	Relative eye separation
Relative forehead size	0.587*	–0.269	0.099	0.196	–0.157
Relative eye size		–0.315	0.480*	–0.157^(^* ^)^	0.202
Relative nose size			–0.204	0.393	0.146
Eye height/width ratio				–0.167	0.242
Nose height/width ratio					–0.462*

Following Benjamini adjustments (Benjamini & Hochberg 1995; Benjamini & Liu 1999) for 15 tests, and using a False Discovery Rate (FDR) of 0.1;

* Significant correlations;

(*) correlations only significant without adjustments.

Similar to eye shape, the average nose height/width ratio was 0.760, indicating wider-than-tall, oval-shaped noses for most dogs. But, unlike relative eye size, relative nose size was not associated with nose shape, as described by this ratio. However, relative eye separation was inversely correlated with nose height/width ratio; those breeds with more widely separated eyes also had smaller nose height/width ratios, meaning that their noses were flatter and wider (see Table 2).

**Figure 3.**
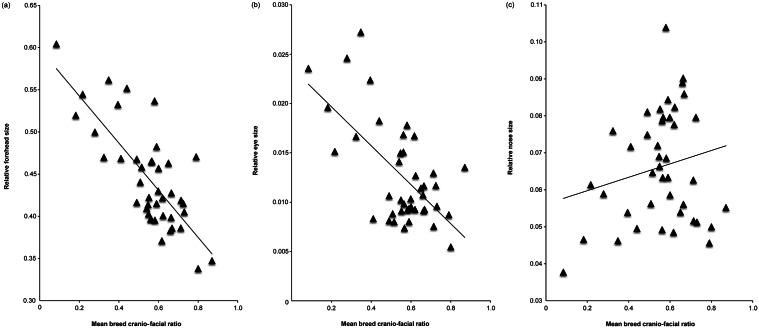
(a) Mean breed cranio-facial ratio (CFR; Packer et al. 2015a) plotted against relative forehead size, (b) mean breed cranio-facial ratio plotted against relative eye size (aperture area) and (c) mean breed cranio-facial ratio plotted against relative nose size. Smaller CFR values indicate a relatively shorter-muzzled, more brachycephalic cranial morphology.

**Figure 4.**
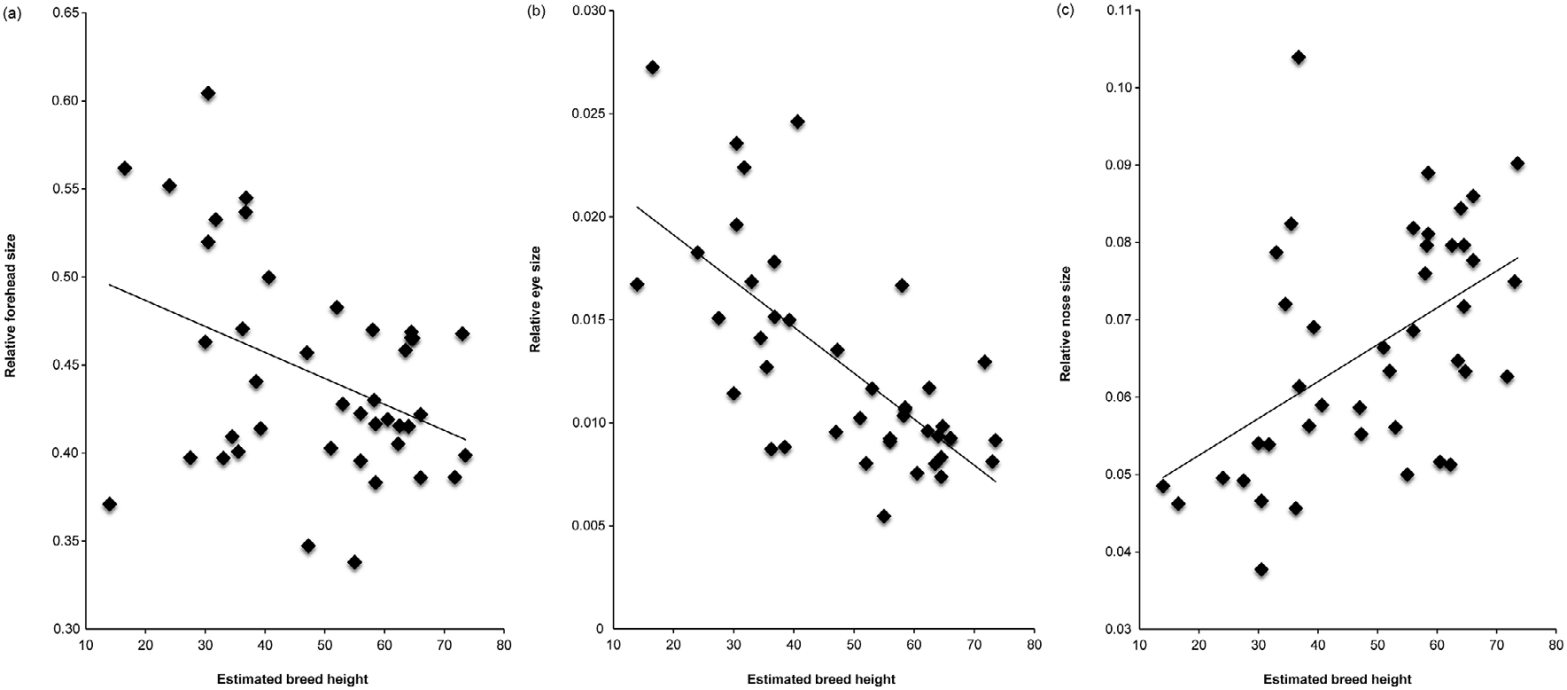
(a) Estimated breed height at withers (The Kennel Club 2021) plotted against relative forehead size, (b) estimated breed height plotted against relative eye size (aperture area) and (c) estimated breed height plotted against relative nose size.

**Figure 5.**
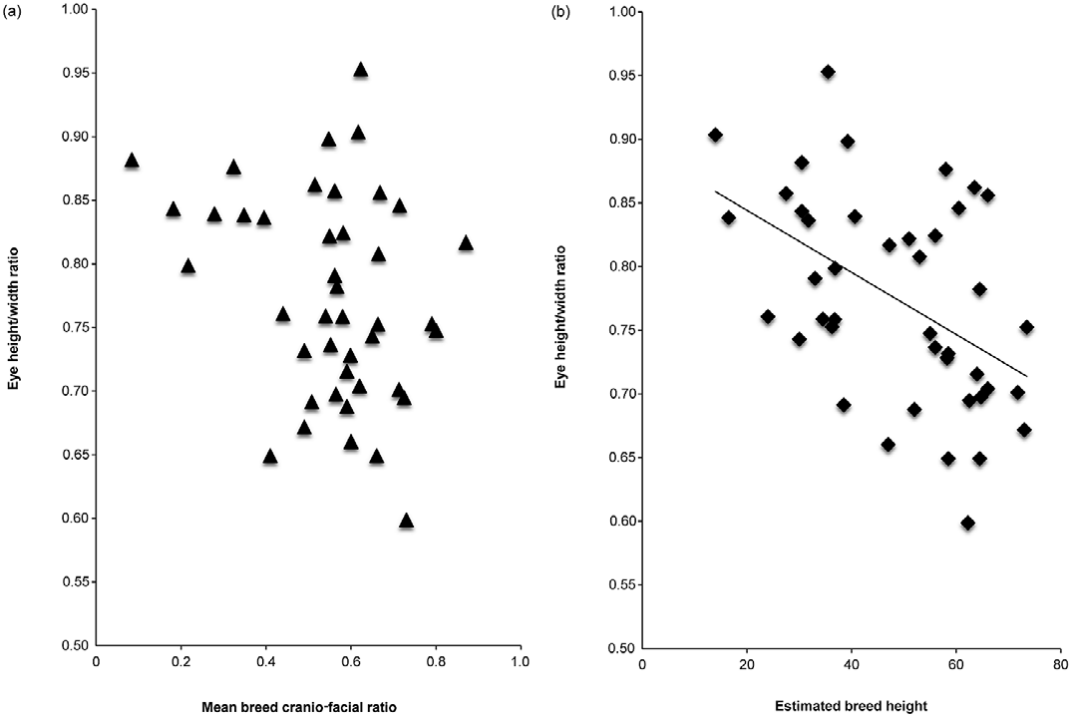
(a) Mean breed cranio-facial ratio (CFR; Packer et al. 2015a) plotted against eye height/width ratio and (b) estimated breed height at withers (The Kennel Club 2021) plotted against eye height/width ratio. Higher eye height/width ratio values indicate taller, more rounded eye shapes (the mean eye shape across the whole sample is a wider-than-tall, almond shape) and smaller CFR values indicate a relatively shorter-muzzled, more brachycephalic cranial morphology.

The results of multiple linear regressions, using relative muzzle length (Model 1 - CFR; Model 2 - CI) and estimated breed height (at withers) as predictor variables, are summarised in Table 3. Shorter-muzzled breeds had relatively larger forehead sizes and relatively larger eyes, but not significantly smaller noses (Figure 3). Smaller breeds (i.e. with lower estimated height at the withers) had relatively larger eyes and relatively smaller noses, but they did not have significantly larger foreheads (Figure 4). However, estimated breed height was significantly associated with eye height/width ratios, with smaller dogs having relatively taller, rounder eyes (Figure 5). Shorter-muzzled breeds did not have rounder eyes or rounder noses. Relative eye separation was not associated with estimated breed height, and not significantly associated with relative muzzle length following Benjamini adjustments for multiple testing (Benjamini & Hochberg 1995; Benjamini & Liu 1999).

**Table 3.** Multiple linear regression results for mean breed fronto-facial ‘kindchenschema’ measures, using relative muzzle length and estimated body height as predictors

Relative forehead size					
Model 1	*B(SE)*	*β*	Model 2	*B(SE)*	*β*
*Constant*	0.613 (0.024)		*Constant*	0.331 (0.043)	
*Breed CFR*	–0.0262 (0.039)	–0.718*	*Breed CI*	0.002 (0.000)	0.606*
*Breed height*	–0.001(0)	–0.134	*Breed height*	–0.001 (0.000)	–0.208
	*R^2^ = 0.603*			*R^2^ = 0.487*	

Relative eye size					
Model 1	*B(SE)*	*β*	Model 2	*B(SE)*	*β*
*Constant*	0.029 (0.002)		*Constant*	0.017 (0.003)	
*Breed CFR*	–0.014 (0.003)	–0.442*	*Breed CI*	0 (0.000)	0.268^(^* ^)^
*Breed height*	0.000 (0.000)	–0.538*	*Breed height*	0 (0.000)	–0.616*
	*R^2^ = 0.656*			*R^2^ = 0.551*	

Relative nose size					
Model 1	*B(SE)*	*β*	Model 2	*B(SE)*	*β*
*Constant*	0.042 (0.008)		*Constant*	0.050 (0.013)	
*Breed CFR*	0.002 (0.014)	0.018	*Breed CI*	0 (0.000)	–0.087
*Breed height*	0 (0.000)	0.498*	*Breed height*	0 (0.000)	0.478*
	*R^2^ = 0.255*			*R^2^ = 0.261*	

Eye height/width ratio					
Model 1	*B(SE)*	*β*	Model 2	*B(SE)*	*β*
*Constant*	0.919 (0.045)		*Constant*	0.901 (0.071)	
*Breed CFR*	-0.071 (0.073)	–0.144	*Breed CI*	0 (0.001)	0.018
*Breed height*	-0.002 (0.001)	–0.428^(^* ^)^	*Breed height*	–0.002 (0.001)	–0.485*
	*R^2^ = 0.248*			*R^2^ = 0.230*	

Nose height/width ratio					
Model 1	*B(SE)*	*β*	Model 2	*B(SE)*	*β*
*Constant*	0.757 (0.028)		*Constant*	0.731 (0.044)	
*Breed CFR*	–0.022 (0.046)	–0.081	*Breed CI*	0 (0.000)	0.076
*Breed height*	0 (0.000)	0.117	*Breed height*	0 (0.000)	–0.111
	*R^2^ = 0.013*			*R^2^ = 0.013*	

Relative eye separation					
Model 1	*B(SE)*	*β*	Model 2	*B(SE)*	*β*
*Constant*	0.436 (0.017)		*Constant*	0.390 (0.028)	
*Breed CFR*	–0.057 (0.028)	–0.329^(^* ^)^	*Breed CI*	0 (0.000)	0.169
*Breed height*	0 (0.000)	0.257	*Breed height*	0 (0.000)	0.190
	*R^2^ = 0.114*			*R^2^ = 0.023*	

Model 1 uses CFR as a predictor, and Model 2 uses CI as a predictor (note: smaller CFR values, and larger CI values, indicate relatively shorter muzzle size, so effects for these are oppositely signed*).* Following Benjamini adjustments (Benjamini & Hochberg 1995; Benjamini & Liu 1999) for 24 comparisons, and using a False Discovery Rate (FDR) of 0.1;

* Significant correlations;

(*) correlations only significant without adjustments.

## Discussion

It is widely agreed that breed-related brachycephalia in dogs is a serious animal welfare problem (e.g. see Asher *et al.* 2009; Rooney & Sargan 2009; Bateson 2016; Brachycephalic Working Group 2017, 2018; British Veterinary Association 2020a,b; O’Neill *et al.* 2022), yet ownership of such breeds has increased dramatically across many countries in recent years (e.g. American Kennel Club 2020; Australian National Kennel Council 2020; The Kennel Club 2020). Many contemporary researchers and commentators have suggested that the current popularity of brachycephalic breeds of dog can be explained by the appealing, cute, ‘kindchenchema’ ‘look’ of these animals (Young & Bannasch 2006; Packer *et al.* 2019, 2020). But this possibility has never previously been investigated. Smaller mid-face regions and larger, more prominent foreheads in (front-of-face) images of humans and animals tend to be regarded as cute (and have been found to be preferred, and perceived as more baby-like, by adult observers: Gardner & Wallach 1965; Alley 1981; Berry & McArthur 1985; see also Figure 1 above). But whether or not breed-related *sagittal plane* muzzle shortening (i.e. the shorter-muzzled, brachycephalic conformation, in profile) in dogs is also associated with infantile ‘kindchenschema’ features in the *coronal plane* (i.e. the front of the face), has remained an open question.

We hypothesised that relative muzzle shortening across a range of popular dog breeds would be associated with a more infantile look when viewed face-on: a relatively large (taller) forehead, large eyes, small nose, round (taller) eyes and nose, and widely spaced eyes (Sternglanz *et al.* 1977; Hildebrandt & Fitzgerald 1979; Alley 1981; Berry & McArthur 1985; McKelvie 1993; Glocker *et al.* 2009; Hecht & Horowitz 2015). We found that, based on visible facial features depicted in a large sample of internet-sourced photographs, our hypothesis was confirmed in two respects. The shorter-muzzled breeds included in this study had relatively larger (taller) foreheads and relatively larger eyes (i.e. larger eye aperture area, in relation to overall face area), but not smaller noses (Figure 3). Trends towards rounder, less almond-shaped eyes and more widely separated eyes among shorter-muzzled breeds did not reach statistical significance. In sum, the present study was able to confirm that muzzle shortening is associated with some, but not all, aspects of a coronal-plane ‘kindchenschem’ ‘look’ among contemporary dog breeds.

Inclusion of estimated breed height in our models enabled us to conclude that associations found between muzzle shortening and forehead and eye aperture area could not be simply explained by the small size of some short-muzzled breeds. Indeed, although many popular, brachycephalic breeds are also small dogs (e.g. Pug, Chihuahua), this type of facial conformation can also be found in larger breeds (e.g. Boxer, Bull mastiff). Furthermore, the sample considered here showed no (inverse) correlation between breed height and CI (Georgevsky *et al.* 2014), and only a small positive correlation between height and CFR (Packer *et al.* 2015b).

### Why is muzzle-shortening associated with fronto-facial features?

The dataset used here describes only the ‘look’ of different breeds, from photographs, not their underlying, three-dimensional structure (i.e. skeletal and soft-tissue composition), so it is not possible to provide a definitive anatomical explanation as to why muzzle shortening in the sagittal plane, and the coronal-plane features of forehead size and eye size, are associated across the breeds studied here. Additional investigations of live dogs (or cadavers) will be needed to document any morphological relationships among different planes of the canine head, and to potentially link these with key genetic mutations (Young & Bannasch 2006; Drake & Klingenberg 2010; Parr *et al.* 2016; Curth *et al.* 2017). Nevertheless, it is possible to speculate on the human factors associated with why the ‘cute’, ‘kindchenschema’ features of sagittal-plane muzzle shortening, and the relatively larger eye and forehead sizes seen in the coronal plane, appear to co-occur across breeds.

First, it is possible that brachycephalic dogs have been selected by humans to look cute and appealing, perhaps unconsciously, to fulfil or enhance a desire for nurturance among owners. If so, then this domestic selection pressure may have acted across multiple morphological features, including eye size, forehead size and muzzle length. Such a scenario could be expected if certain types of dog have been used specifically to function as infant- or child-like companions; to look cute and to stimulate nurturant feelings and behaviour in their owners (Serpell 2002; Waller *et al.* 2013; Borgi & Cirulli 2016; Serpell 2019; see also Serpell 1996). If this is the case, then it is possible that genetic control of each of these features may be wholly or partially independent, having been modified together simply as a result of having been selected together (for discussions of modularity in cranial features of the dog, see: Parr *et al.* 2016; Curth *et al.* 2017). There may, therefore, be future possibilities for breeding dogs in whom some appealing, infantile features could be maintained, while clinically problematic muzzle shortening in the sagittal plane of the skull is selected against. Of course, this raises the wider question of exactly how much facial features can be genetically modified to achieve some degree of ‘kindchenschema’ effect, whilst also maintaining (or preferably, maximising) conformational health. In other words, *how cute is too cute* (Packer 2013)? More specifically, for example, how short can a dog’s muzzle be before brachycephalic obstructive airway syndrome (BOAS) occurs (Packer *et al.* 2015b), how large can eyes be before clinical pathologies such as corneal disease develops (Packer *et al.* 2015a), and how high or domed can a forehead be before neurological disorders, such as syringomyelia, emerge (Mitchell *et al.* 2014)?

A second possibility is that only a single facial feature, or subset of features, has been selected for by breeders, and the others have also changed as a result of morphological linkage, i.e. by a process of co-selection. For example, a number of contemporary brachycephalic breeds are thought to have originated as fighting dogs (e.g. Bulldogs; Ellis *et al.* 2009; American Kennel Club 2017). Those individuals with shorter muzzles may have initially been favoured for breeding because they conferred an advantage in the pit or ring (Young & Bannasch 2006). So, if dogs with short muzzles were selectively bred for this (or other) reasons, it is possible that as their muzzles became shorter, this change also gave rise to a wider suite of ‘kindchenschema’-like features in the coronal plane of their faces. One version of this possibility has been expressed as the neotenisation hypothesis; the proposal that selection for one particular morphology or ‘look’ may have affected development of the whole cranium. For example, some of the genetic mutations harnessed to produce brachycephalic dogs may influence head and face morphology via the entire developmental progression of cranial growth (Pittenger & Shaw 1975; Todd *et al.* 1980; Frank & Frank 1982; Coppinger & Smith 1983; Jones 1995; although see also Drake 2011). Currently, and while remaining a popular view, this hypothesis remains unproven. However, if such a co-selection account is correct, then any amount of future re-breeding may fail to separate the cute, coronal-plane ‘kindchenschema’ appearance from clinically problematic muzzle shortening.

Sources of variation in skull morphology across contemporary dog breeds appears to rely on the contributions of mutations from many different quantitative trait loci (Schoenebeck *et al.* 2012; Bannasch *et al.* 2020). For example, Bannasch *et al.* (2020) identified strong genome-wide associations for brachycephalic head type on the Cfa1 gene, using comparisons of strongly brachycephalic, compared with non-brachycephalic dogs. Others have found additional mutations to be associated with brachycephalic breeds, including in the gene BMP3, which has been shown to function in cranial development in a wide range of species (Schoenebeck *et al.* 2012), and a mutation in the DISHEVELLED2 (DVL2) gene, apparently associated with vertebral and skull malformations in Bulldogs, French bulldogs and Boston terriers (Mansour *et al.* 2018; Niskanen *et al.* 2021). Reduced muzzle length is a breed-defining characteristic in most of these dogs, as can be seen from kennel club breed standards (Asher *et al.* 2009; Packer *et al.* 2015b), and it appears that the degree of muzzle shortening in these breeds has increased across the past 50 to 100 years (Young & Bannasch 2006; see also Knowler *et al.* 2019; Skipper 2021). But the relative contribution of all of these different genes to the coronal-plane ‘kindchenschema’ facial features are, as yet, unexplored. It is possible that further investigations of sagittal- and coronal-plane feature associations will find variance according to breed clades and their associated mutations.

### Differences in relative muzzle length, as measured by CFR and by CI

Differences between breeds in the precise forms that muzzle shortening can take is highlighted by differences between the CFR and CI measures. It has previously been noted that muzzle shortening across dog breeds does not represent a single morphological process, with the sagittal shapes of different short-muzzled breeds varying considerably (e.g. Georgevsky *et al.* 2014; Geiger *et al.* 2021). Perhaps the most dramatic illustration of this can be seen when two brachycephalic dogs, the Chihuahua and the Pug, are compared. The Chihuahua (especially the ‘apple-headed’ sub-type) has a small but domed and protruding forehead, while the Pug’s forehead profile is an almost continuous rearward slant from nose to ears. Measuring relative muzzle lengths across breeds using the CFR and CI methods yields highly correlated datasets but the two methods do differentiate some morphological variation, including this contrast between the Pug and the Chihuahua. Specifically, the soft-tape method of the CFR captures the roundedness of the Chihuahua forehead, effectively reducing the ‘relative muzzle length’ measure as a result (i.e. using the CFR method exaggerates the estimation of muzzle shortness of Chihuahuas, in comparison with the CI). In the current analyses, the shorter-muzzled breeds were found to have relatively large eye aperture areas, but this significant finding related only to muzzle length measured by CFR (Packer *et al.* 2015b). It is possible, therefore, that the forehead doming in some breeds, such as the Chihuahua, plays an additional role in determining this association. For future research (e.g. both further ‘kindchenschema’ investigations, and morphological studies designed to seek associations with clinical disease), additional measures of muzzle shape (e.g. cranio-facial angle; Regodon *et al.* 1993) and more specific measures of forehead doming, in addition to relative muzzle length, may prove informative (see also Brehm *et al.* 1985).

### Breed height and ‘kindchenschema’ features

Estimated breed height was positively correlated with relative nose size (smaller breeds had smaller noses, relative to overall face size), and inversely correlated with relative eye aperture area (smaller breeds had larger eyes, relative to overall face size) (Figure 4). Smaller breeds also had somewhat rounder, less almond-shaped eyes than larger breeds (Figure 5). However, no associations were found between breed height and relative forehead size. In sum, short-statured dog breeds showed some ‘kindchenschema’, ‘cute’ features in the coronal plane of the face, but these differed, to a degree, from those seen among short-muzzled dogs.

As with muzzle shortening, it is possible to speculate about the origins of these facial-feature linkages in the selective breeding of relatively small and large breeds of dog. Of all domesticated animals, dogs are notable for their wide variability of stature, with selection for smaller and shorter breeds being associated with a number of breed clades and (original) breed functions (Bannasch *et al.* 2020; Geiger *et al.* 2021). But while the small size of some short-limbed dogs can be explained by the effects of a single-gene mutation (e.g. FGF4 in Dachshunds; Parker *et al.* 2009), breed size across much of the contemporary dog population is a smoothly continuous variable that is likely to have multiple genetic sources (Bannasch *et al.* 2020). We can hypothesise that selecting for small, ‘cute’ lapdogs may, like breeding for shortened muzzles, have had direct (independent) effects on a range of features, and/or linked effects across genetically associated features. Relatively large eyes, for example, which were associated in the present sample with both muzzle shortening and short stature, may represent concurrent selection for a known ‘kindchenschema’ feature (i.e. one that elicits preference and cuteness attributions in observers; Kringelbach *et al.* 2016). However, it is also possible that smaller dogs have relatively large (and rounder) eyes because of a mismatch between the miniaturisation of the skeleton (and in particular the eye socket) and of the soft tissue of the eyes and themselves, resulting in larger, more protruding eyeballs (Geiger *et al.* 2021). This may be particularly distinctive of domestic breeding (i.e. as compared with miniaturisation resulting from natural selection), resulting from the rapid selection and crossing that some breeds have undergone (e.g. see Parker *et al.* 2017), and the relative lack of functional selection pressures (Drake & Klingenberg 2010). For example, among wild species where body size reductions have occurred (e.g. as a result of island miniaturisation; Lomolino 2005), similar mismatches between skeletal size and eye size do not appear to occur, although extensive research in this area is lacking. Among contemporary dogs, whether certain breed clades show more or less skeletal/soft tissue disproportionality than others, and whether this is associated with greater/lesser vulnerability to particular clinical disorders and consequent welfare problems, remain important questions for future morphological research (e.g. Parker *et al.* 2017).

Previous research (McGreevy *et al.* 2004) has shown that the volume of the canine eye varies with dog size more than was first thought. The authors reported a correlation between skull and eye size, refuting previous suggestions that eye radius in dogs is a fairly constant 11 mm (Coppinger & Schneider 1995), but that instead it ranged from 9.6 to 11.6 mm. This supports the suggestion that large dogs have large eyes (Peichl 1992). The same study (McGreevy *et al.* 2004) confirmed that eyelid apertures vary considerably in their orientation within the skull, with the angle of orientation, sub-tended by their midlines, varying from 10° (very frontally placed eyelid apertures) to 50° (more laterally placed eye apertures). These angles were found to correlate with the skull length and cephalic index and, to a lesser degree, skull width. However, a regression analysis revealed that the predictor for eyelid apertures’ orientation was cephalic index and not skull width and length.

### Relative eye separation

An additional facial feature considered here was relative eye separation. Although the appearance of widely separated eyes (hypertelorism) has not been extensively studied as a ‘kindchenschema’ feature, it is consistent with the infantile profile of a relatively enlarged cranial vault. In addition, a recent experimental study on the effects of ‘kindchenschema’ features on people’s judgements of animal photographs found that eye separation can increase preferences for manipulated images of dogs’ faces (Hecht & Horowitz 2015). However, in the current study, breed-level variation in relative eye separation was not significantly associated with shorter relative muzzle lengths (or smaller breed height; Table 3). This was somewhat surprising, given that wide eye-placement has been found to be linked with a key mutation present in a number of brachycephalic dog breeds. Mansour *et al.* (2018) conducted whole genome sequencing across 21 breeds, identifying a shift mutation in the WNT pathway gene DVL2 in three of those breeds: Bulldogs, French bulldogs and Boston terriers. In our dataset of 42 breeds, the top ten with greatest relative eye separation were: Boston terrier, Great Dane, Bull terrier, English springer spaniel, French bulldog, Bulldog, Boxer, Weimaraner, Gordon setter, Dachshund (miniature). So, although a number of these breeds showing the greatest eye separation are brachycephalic (and the three known DVL2 carrier breeds are in the top ten), they were also intermixed with others with much longer muzzles. While again noting that relative eye separation in the current study was based on measurement of photographs, rather than actual skeletal measurements, this finding suggests a mixed provenance of the eye separation trait, with the possibility that several different genes, and consequent skeletal morphologies, influence variation in eye separation across breeds, and that not all of these are associated with brachycephalia.

The only other facial feature that was associated with relative eye separation in the present study was nose height/width ratio; specifically, breeds with more widely separated eyes had relatively wider, more oval-shaped noses in the sample of images measured here (Table 2). This finding is likely to be indicative of increased eye separation being associated with a more general widening of the midface region of the skull. This is not unexpected; in humans diagnosed with orbital hypertelorism, for example, associated conditions include median facial clefting, resulting in a wide, flattened nose area (Benacerraf *et al.* 2019).

### Animal welfare implications

A number of commentators have attributed the current, extreme popularity of brachycephalic dogs to the attractive qualities of their ‘cute’, ‘kindchenschema’, facial features (e.g. Tuan 1984; Serpell 1996; McGreevy *et al.* 2013; Packer *et al.* 2017; Sandøe *et al.* 2017; O’Neill *et al.* 2022), and the present findings are not inconsistent with that view. The hypothesis that selection for nuturance is an important driving force in the development of companion dog ownership (and pet-keeping more broadly) is certainly one that must be taken seriously (Serpell 1996); if companion dogs are selected across time to have increasingly cute looks (both within breeds, and between breeds in the sense of increased breed-share of the whole owned-dog population), then in the future, welfare problems with brachycephalia and other associated morphologies are destined to grow. Nevertheless, many details of this nurturance hypothesis are still to be fleshed out, and many other factors may be at play in determining the popularity of brachycephalic breeds (e.g. Bognár *et al.* 2021). Moving forward to consider the future popularity of cute-looking, short-muzzled dogs, it is important to note that the ‘kindchenschema’ effect shows some cultural dependence, with owners in different countries varying in their preference for brachycephalic pets (see Farnworth *et al.* 2018 regarding cat breed preferences; see also Archer & Monton 2011 regarding the effects of pet ownership). These findings, combined with the fact that many non-brachycephalic dogs also remain highly popular as pets (Brincat *et al.* 2022), indicate that people’s preferences do vary, and ‘kindchenschema’ features are far from being the only determinant of breed preference. Cultural fashions come and go, so something that seems like a permanent change or progression at one point in history may be only a short-lived trend (Herzog & Elias 2004; Herzog *et al.* 2004; Herzog 2006; Ghirlanda *et al.* 2013, 2014; Skipper 2021). Further social and psychological research is needed to establish how important facial ‘kindchenschema’ features are to prospective dog owners, and how an understanding of the clinical and welfare consequences of extremely short muzzles may be used to modify pet ownership and breeding practices in the future.

## Supplementary material

For supplementary material accompanying this paper visit https://doi.org/10.1017/awf.2022.6. click here to view supplementary material
