# Supplementary material for: That brachycephalic look: Infant-like facial appearance in short-muzzled dog breeds
Source: Anim Welf. 2023 Jan 26;32:e5. doi: 10.1017/awf.2022.6 (PMC10936394; doi:10.1017/awf.2022.6)
Supplement: Supplementary file 1 [file S0962728622000069sup001.pdf]

Appendix 1

Figures S1 to S6 detail the means and standard deviations for all six facial features measured among the exemplar photographs of all 42 breeds studied here. Twenty exemplar photographs were included for each breed; it can be seen here that within-breed variation is generally low for all features. Most inter-breed variation was seen in relative eye size, and least in relative nose shape (nose height/width ratio) and relative eye separation. It was also noted that some breeds (Pug, American cocker spaniel, Cocker spaniel) showed greater relative feature size/shape variation than others.

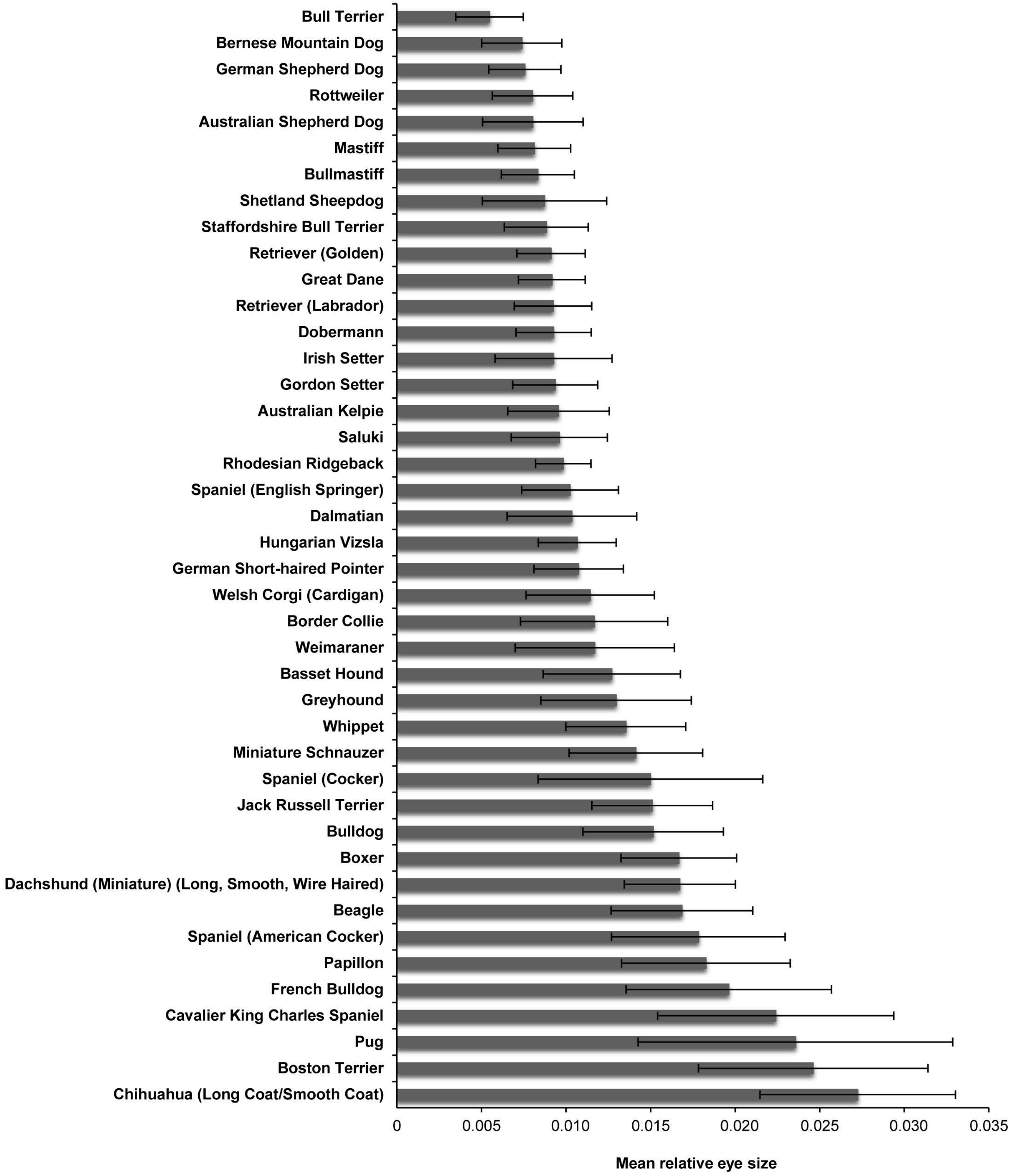

Figure S1 Means and standard deviations of relative eye size (eye aperture area/total face area) for 20 exemplar photographs each of 42 breeds.

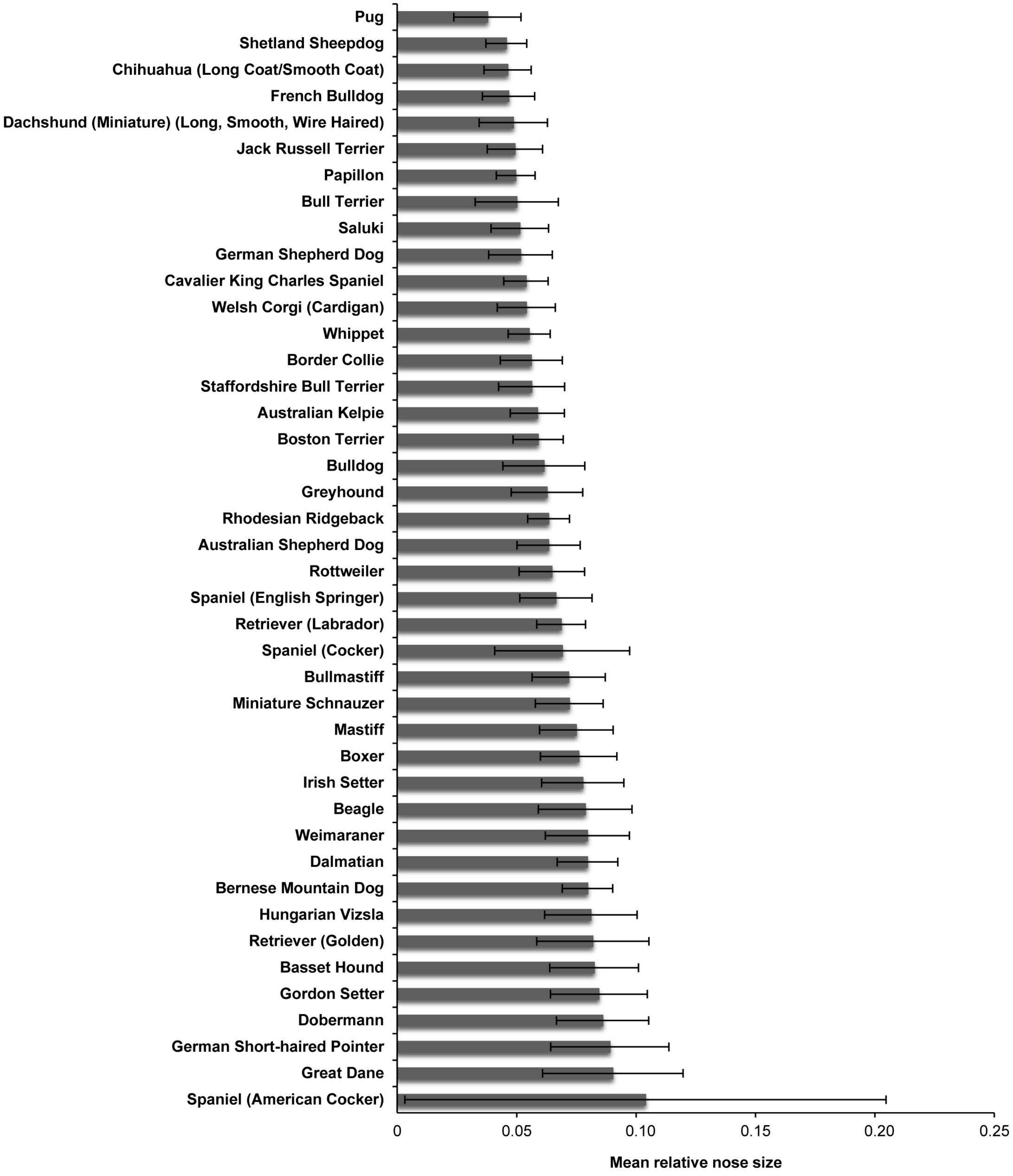

Figure S2 Means and standard deviations of relative nose size (nose area/total face area) for 20 exemplar photographs of 42 breeds.

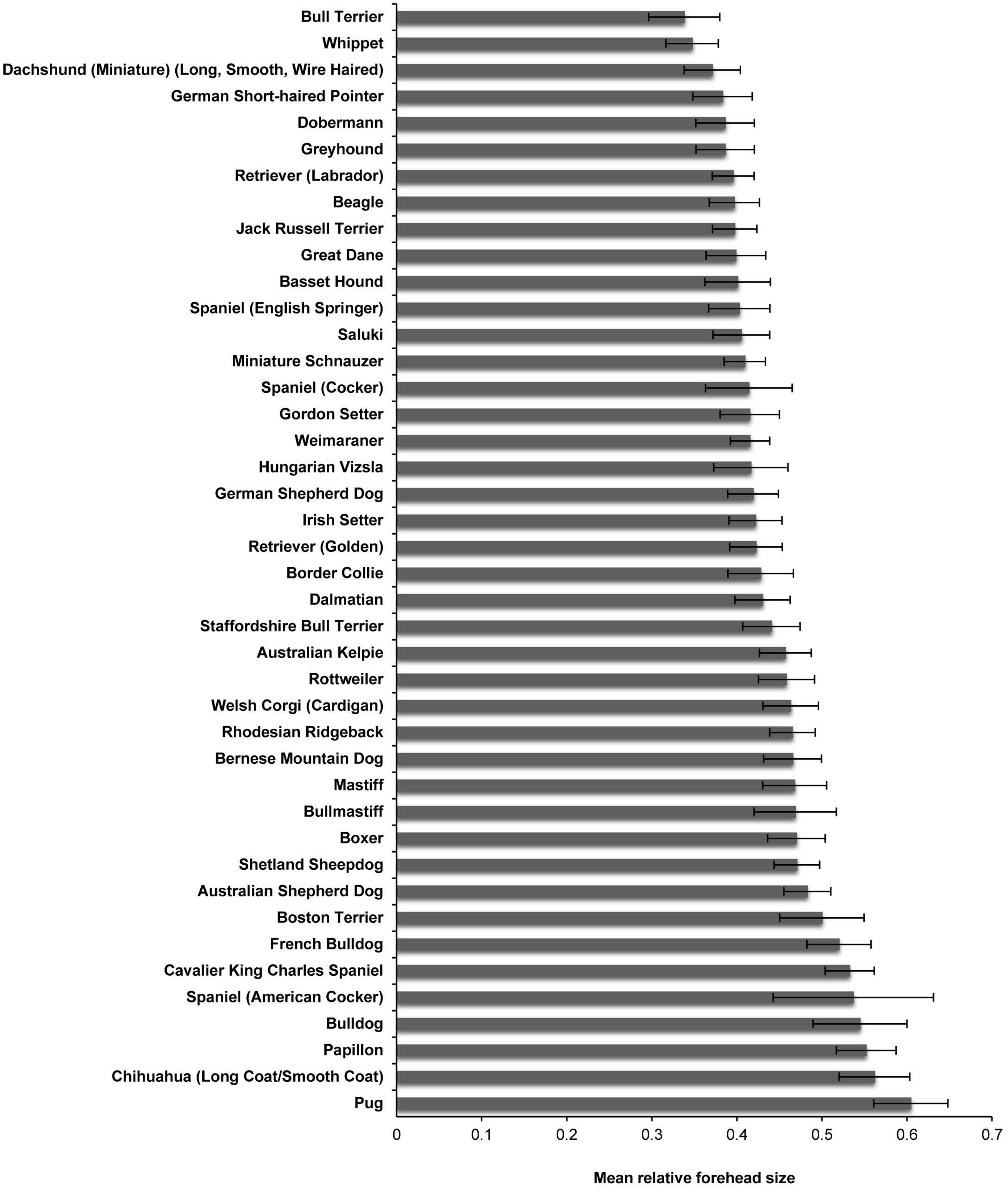

Figure S3 Means and standard deviations of relative forehead size (forehead area/total face area) for 20 exemplar photographs of 42 breeds.

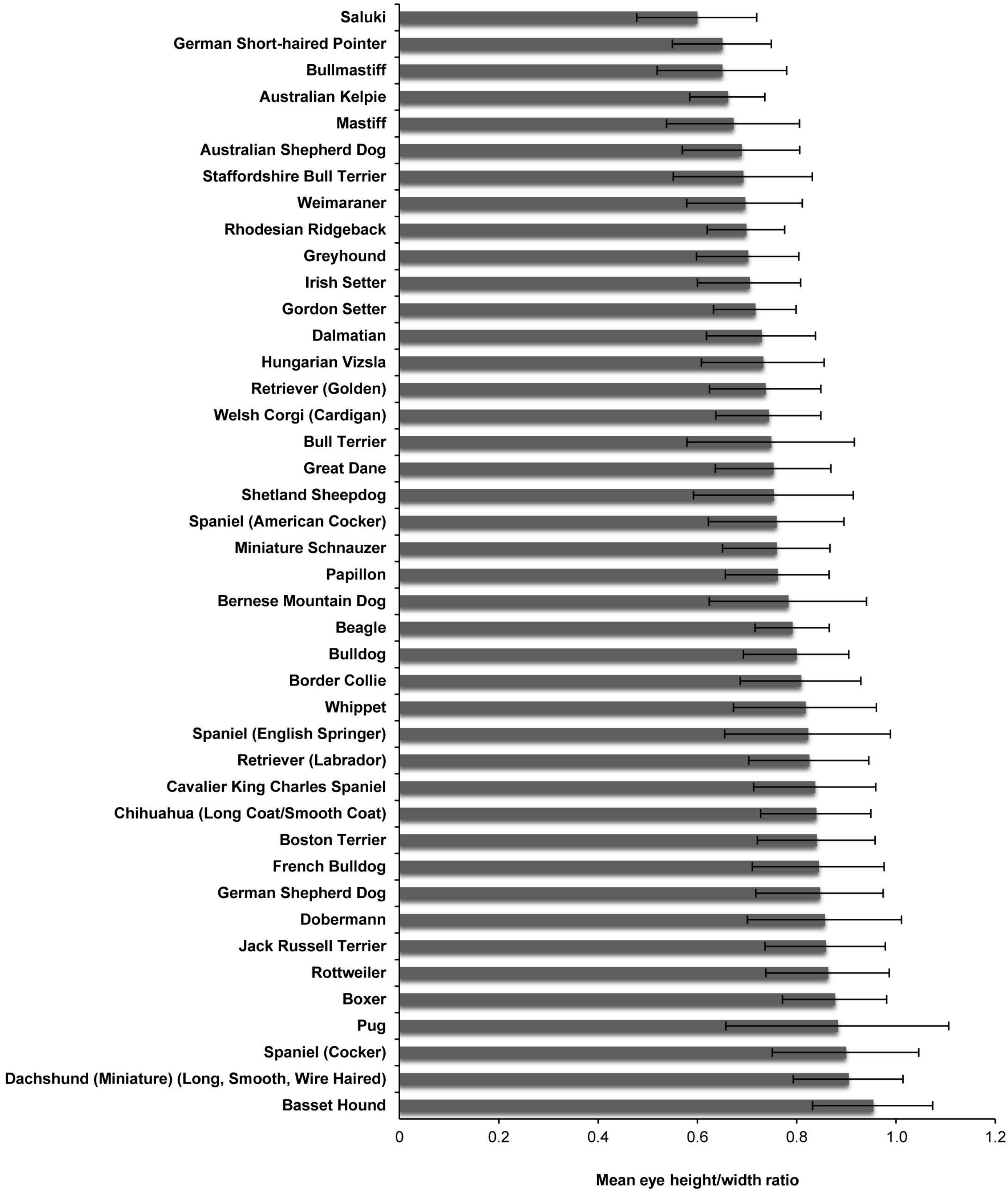

Figure S4 Means and standard deviations of eye height/width ratio for 20 exemplar photographs of 42 breeds (larger ratios represent relatively taller, rounder eyes).

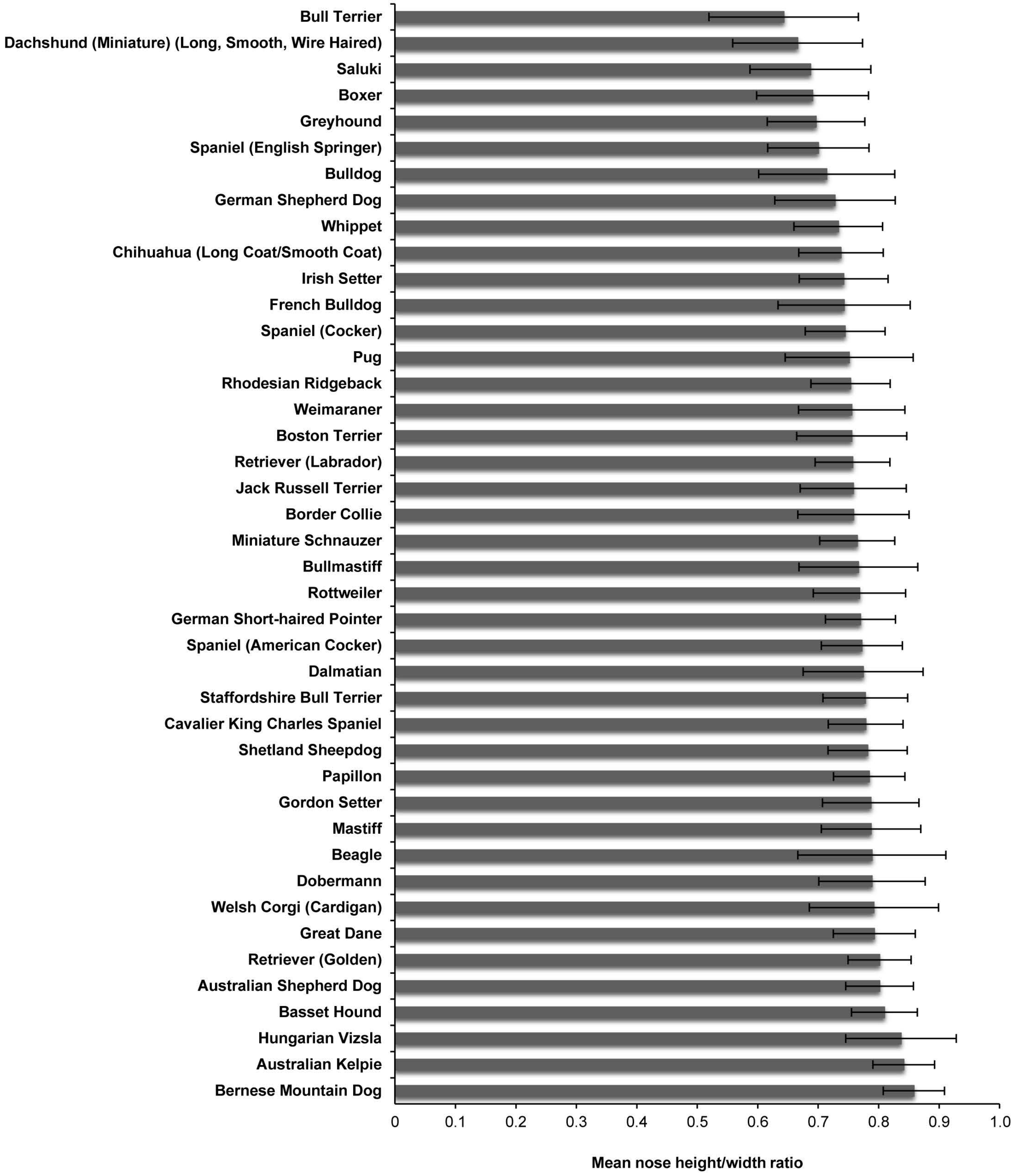

Figure S5 Means and standard deviations of nose height/width ratio for 20 exemplar photographs of 42 breeds (larger ratios represent relatively taller, rounder noses).

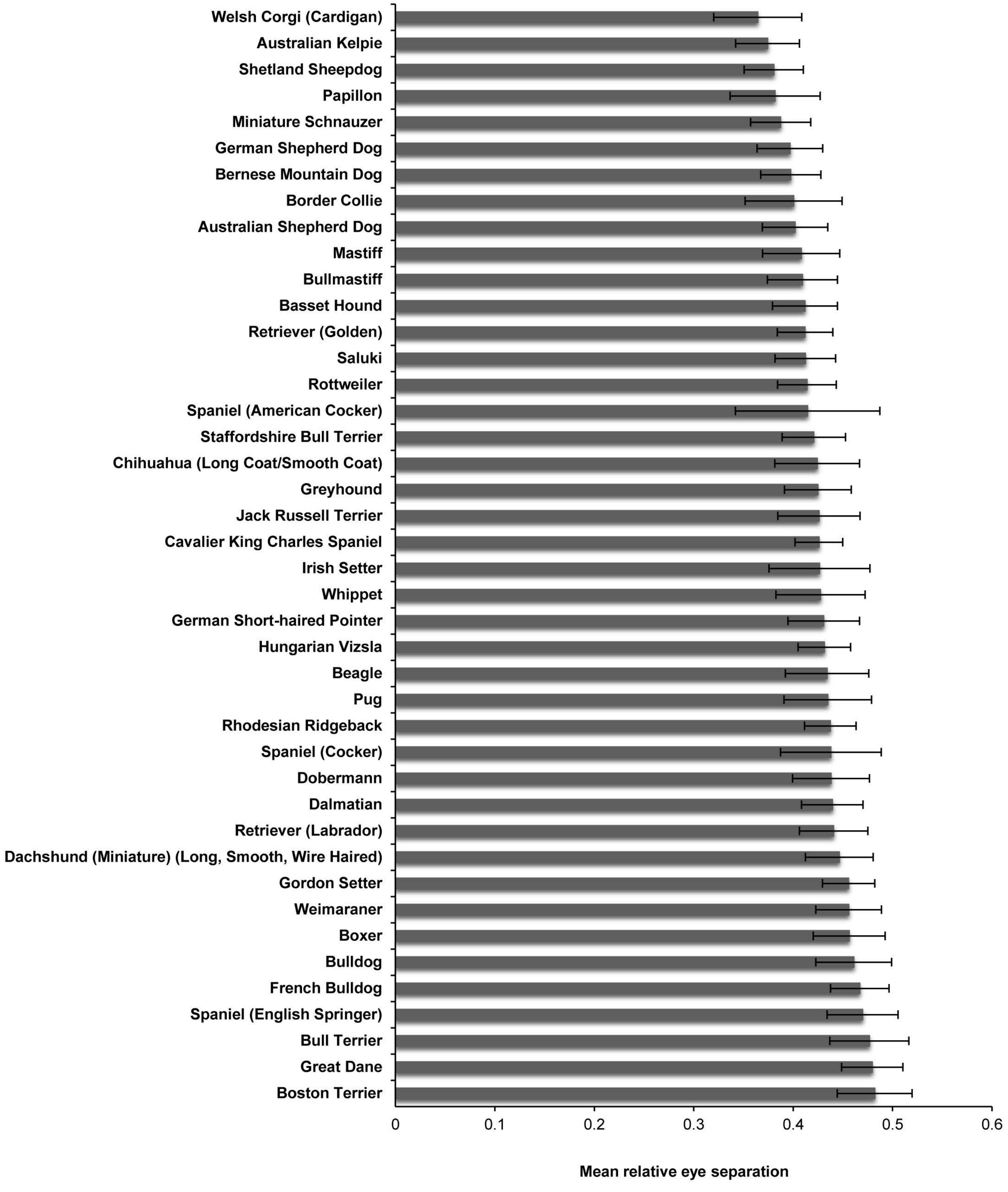

Figure S6 Means and standard deviations of relative eye separation (distance between centre of each eye/face width) for 20 exemplar photographs of 42 breeds.
